# Fluorine-Assisted Self-Assembly Triggers Peculiarities in Molecular Dynamics of a Polar Glass-Former

**DOI:** 10.1021/acs.jpcb.5c00410

**Published:** 2025-05-22

**Authors:** Zaneta Wojnarowska, Mateusz Dulski, Jie Shen, Beatrice Ruta, Martin Rosenthal, Marian Paluch

**Affiliations:** † Institute of Physics, the University of Silesia in Katowice, 75 Pułku Piechoty 1A, 41−500 Chorzów, Poland; ‡ Institute of Material Sciences, the University of Silesia in Katowice, 75 Pułku Piechoty 1A, 41−500 Chorzów, Poland; § Institut Néel, Université Grenoble Alpes and Centre National de la Recherche Scientifique, 25 rue des Martyrs − BP 166, 38042 Grenoble, France; ∥ ESRF, 71, Avenue des Martyrs, CS40220, 38043 Grenoble Cedex 9, France; ⊥ Department of Chemistry, 26657 KU Leuven, Celestijnenlaan 200f, 3001 Leuven, Belgium

## Abstract

Condensed matter physics has long struggled to obtain a comprehensive picture of the liquid-glass transition. Consequently, over the years, universal manifestations of glassy and supercooled dynamics have been established, including a correlation between the static dielectric constant (Δε) and relaxation stretching (β_KWW_), as well as β_KWW_ and Kirkwood correlation factor (*g*_K_), or deviation degree from Arrhenius behavior of structural relaxation times quantified by dynamic fragility (*m*_P_). Herein, we report a simple, highly polar liquid that breaks all of these rules established for glass-forming liquids. We show that the fluorine-assisted self-assembly, confirmed by temperature-dependent Raman and XRD measurements, brings peculiarities in relaxation dynamics, that is, extremely low dielectric strength, Debye-like shape of dielectric permittivity spectra, *g*_K_ much below unity, and enormous acceleration of structural relaxation times and viscosity at *T* = *T*_g_ + 20 K. All these peculiarities reveal a strong effect of molecular self-assembly on the dynamics of glass-forming systems.

## Graphical abstract

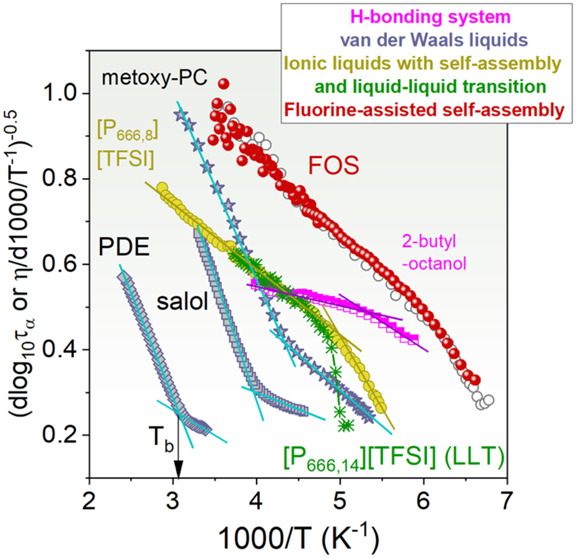

## Introduction

Nearly five decades of research on various glass-forming systems, including polymers, low-molecular-weight organic and inorganic compounds, ionic liquids, and metallic materials,^1–3^ confirmed Turnbull’s assertion, “Nearly all materials can, if cooled fast enough and far enough, be prepared as amorphous solids”.^4^ Furthermore, a quasi-universal description of glass formation was formulated. It has been well-established that the volume (V) and enthalpy (H) of all glass-formers, with the exception of water, decrease continuously on cooling with a characteristic intersection splitting the metastable supercooled liquid (SL) from the nonequilibrium glass (G) at the liquid-glass transition temperature (*T*_g_).^5,6^ At the same time, molecular motions slow substantially from the time scale of picoseconds in a low-viscosity equilibrium liquid state (around melting point *T*_m_) up to hundreds of seconds below *T*_g_. Then, the liquid structure becomes ‘frozen’ on the laboratory time scale with a viscosity of 10^12^ Pas and mechanical properties of a solid state.^7^ There are theories presuming that the significant temperature sensitivity of relaxation times or viscosity close to *T*_g_ is predominantly a kinetic phenomenon^8,9^ and others that propose a thermodynamic (entropic) origin. However, regardless of the paradigm, it is undeniable that viscous liquids are structurally and dynamically heterogeneous and do not relax uniformly but exhibit fast and slow regions.^10^ Considering the temperature evolution of dynamic quantities, the supercooled liquids can be classified into ‘strong’ or ‘fragile’ along the Angell scale.^11^ The viscosity and relaxation times of the former behave in nearly Arrhenius fashion on cooling, whereas fragile liquids (the vast majority of materials) show substantial deviations from linear behavior usually described by the phenomenological Vogel–Fulcher–Tammann (VFT) expression, $\eta =\eta_{0}\;\exp\left(\frac{DT_{0}}{T-T_{0}}\right)$ , where other dynamic quantities can replace viscosity η and strength parameter D is a measure of non-Arrhenius behavior.^12^ Despite its simplicity, the VFT equation works well, at least eight decades after the liquid-glass transition.

A second essential fingerprint of supercooled liquid is the nonexponential nature of the structural (α) relaxation.^13^ An asymmetrical broadening of relaxation spectra is quantified by the high-frequency power law ν^–β^ with β ≤ 1. When the relaxation stretching is probed by depolarized dynamic light scattering (DDLS) or mechanical spectroscopy, a generic value β ≈ 0.5 is observed for various low-molecular glass-formers.^14^ On the other hand, exponent β of dielectric permittivity spectra *ε*″(ω) recorded at *T* > *T*_g_ usually spans the range from 0.5 to 0.8, depending on molecule polarity. Specifically, the larger the dielectric relaxation strength Δε = ε_s_ – ε_∞_ ∼ Nμ^2^ (where ε_s_ and ε_∞_ are the zero and high-frequency limits of the dielectric permittivity), reflecting the magnitude of dipole moment, the narrower the α-loss peak. This correlation has been successfully verified for more than 88 van der Waals glass-formers^15^ and rationalized by strong dipole–dipole interactions that increase the harmonicity of the intermolecular potentials, which in turn affect the distribution of relaxation times and, thus, the relaxation stretching. So far, the Δε­(β) relation has not been satisfied only for monohydroxy alcohols that reveal strong dipole–dipole interactions within the H-bonding network. For these systems, two contributions to the dielectric ε″(ω) spectra are usually observed: Debye-like process originating from cross-correlations between dipoles and self-correlation revealing the same characteristics as the relaxation process obtained in DDLS measurements.^14^ From this perspective, associated liquids can be considered an exception from the universal features of the metastable supercooled liquid.

Herein, we report the first example of a glass-forming liquid of high dipole moment that is characterized by a relatively narrow dielectric relaxation spectrum and peculiar evolution of structural relaxation times and viscosity in the close vicinity of the liquid-glass transition. These observations were discussed in terms of molecular self-assembly, as confirmed by temperature-dependent Raman and XRD measurements.

## Methods

### XRD

The sample is transferred into a sealed capillary glass tube (diameter 2 mm), and the X-ray detects the central portion of the sample. The X-ray energy is 12 keV, with detectors positioned at two locations for small-angle X-ray scattering (SAXS) (Pilatus1M) and wide-angle X-ray Scattering (WAXS) (Pilatus300kw), at distances of 1.44 and 0.28 m from the sample, respectively. Signals are detected simultaneously, with a data acquisition frequency of 30 s per frame. Temperature control is achieved using a cryostream system with cooling and heating rates of 1 K/min. Signals from the capillary glass tube are independently collected to serve as background signal subtraction. The one-dimensional diffraction intensity is obtained by azimuthal integration of diffraction patterns by using the pyFAI software.

### Differential Scanning Calorimetry (DSC)

Calorimetric experiments of the studied FOS were performed by means of a Mettler Toledo DSC1STAR system equipped with a liquid nitrogen cooling accessory and an HSS8 ceramic sensor (a heat flux sensor with 120 thermocouples). During the experiments, the flow of nitrogen was kept at 60 mL min^–1^. Enthalpy and temperature calibrations were performed by using indium and zinc standards. The baseline was constructed as a straight line from the onset to the endpoint. A dedicated software, Mettler Toledo DSC1STAR, allows various calculations (onset, heat, peak temperature, etc.) from the original recorded DSC curves.

### Dielectric Measurements

The dielectric measurements at ambient pressure for the studied FOS were carried out over a frequency range from 10^–1^ to 10^7^ Hz by means of a Novo-Control GMBH dielectric spectrometer. The Quattro system controlled the temperature with an accuracy of 0.1 K. During this measurement, the sample was placed between two stainless steel electrodes (diameter = 15 mm). The quartz ring provided the distance between plates.

### Viscosity Measurements

The viscosity was measured employing an ARES G2 Rheometer. In the supercooled liquid region, aluminum parallel plates of diameter 4 mm were used, while the viscosity of the normal liquid state was measured using 25 and 50 mm geometries. The rheological experiments were performed in the frequency range from 0.1 to 100 rad·s^–1^ (10 points per decade) with strain equal to 0.01% in the vicinity of the liquid-glass transition. The strain was increased by 1 order of magnitude with every 10 K. The relative uncertainty of the reported viscosity measurements *u*_r_(η) from calibration, temperature, and pressure control, as well as sample purities, did not exceed 7%.

### Raman Measurements

Temperature-dependent Raman spectroscopy was conducted using a WITec confocal Raman microscope (CRM) α 300R, equipped with a solid-state laser (λ = 457 nm). The laser was integrated into the microscope via a polarization-maintaining single-mode optical fiber with a diameter of 50 μm. The laser beam was focused on the sample through a long-distance Olympus MPLAN objective (50*x*/0.76 NA), while the scattered light was collected through a multimode fiber of the same diameter. Before measurements, the spectrometer’s monochromator, featuring a 600 lines/mm grating, was calibrated using a silicon plate (520.7 cm^–1^). In turn, the sample, due to its low-viscosity nature, was placed in a distinct cylindrical cap and positioned on a THMS600 Linkam stage.

Raman spectrum at room temperature was acquired with a laser power of 20 mW, utilizing ten scans, an integration time of 10 s per scan, and a spectral resolution of 3 cm^–1^. Subsequently, each sample was progressively cooled to 213 K in steps of 10 K. Further, down to 133 K in steps of 2 K, maintaining a cooling rate of 10 K/min and a temperature stabilization accuracy of 0.1 K. At each temperature point, Raman spectra were collected with five scans, an integration time of 10 s per scan, and a resolution of 3 cm^–1^. All acquired spectra underwent postprocessing, including cosmic ray removal and baseline correction, utilizing WITec Project Five Plus software (5.1.1). Finally, the Raman spectra were normalized to elucidate the temperature-dependent changes more clearly.

## Results and Discussion

The chemical structure of the examined fluid 1*H*,1*H*,2*H*,2*H*-perfluorooctyltriethoxysilane (abbreviated as FOS) is presented in Figure 1a. A characteristic feature of the FOS molecule is an eight-atom fluorocarbon chain connected to a silica core. Due to the high electronegativity of fluorine, FOS possesses a relatively high-permanent dipole moment μ = 4.39 D and a rigid fluoroalkyl chain with helical conformation, compared to the all-trans conformation of hydrocarbon. Consequently, in analogy to other highly polar glass-forming systems, significant static dielectric constants and narrow distributions of relaxation times are expected in FOS. Therefore, a Novo-Control analyzer connected to the Quatro temperature controller was employed for dielectric experiments. The representative permittivity loss spectra collected for FOS are shown in Figure 1a. The well-resolved dielectric loss peak ε″(*f*), representing structural (α) relaxation, is clearly visible in the examined frequency window. It moves toward lower frequencies with cooling, which reflects the behavior typical of supercooled liquids. However, a closer inspection of the collected data reveals peculiarities in the dielectric response of FOS. The dielectric loss curves of FOS are very narrow. The KWW function with the stretch exponent β_KWW_ of 0.82 is necessary for proper parametrization of the α-process (see inset of Figure 1b). Furthermore, close to *T*_g_ ε_s_ of FOS (ε_s_ = 7.8) is markedly lower than the dielectric constant reported for other glass-formers of similar polarity.^16^ Consequently, the correlation between β_KWW_ and Δε­(*T*_g_) parameters is not satisfied since the static dielectric constant should be at least 10 times higher (see Figure 1b).

**Figure 1:**
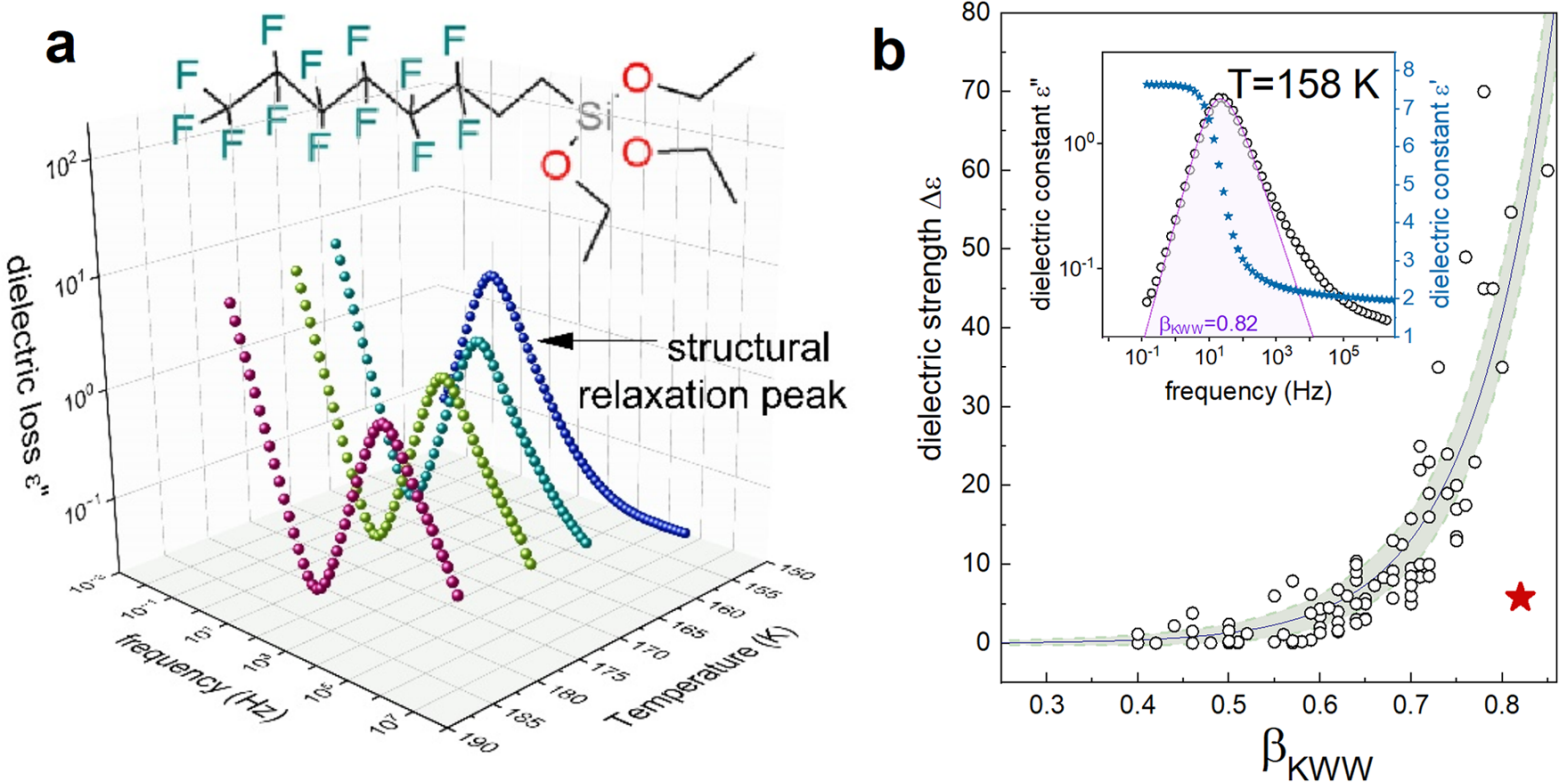
(a) Representative dielectric loss spectra of FOS. The inset represents the chemical structure of the FOS molecule. (b) Dielectric strength of polar glass-formers versus relaxation strength. The red star represents Δε and β_KWW_ values determined for FOS.

To shed light on the physical origin behind these observations, the preferred orientation of neighboring FOS dipoles was examined through the Kirkwood correlation factor, $g_{K}=\frac{9k\varepsilon_{0}M_{\mathrm{mo}l}T}{N_{A}\rho \mu^{2}}\frac{\left(\varepsilon_{s}-\varepsilon_{\infty }\right)\left(2\varepsilon_{s}+\varepsilon_{\infty }\right)}{{\varepsilon_{s}{(\varepsilon }_{\infty }+2)}^{2}}$ , where *k* is Boltzmann’s constant, *M*_mol_ is the molar weight, ε_0_ = 8.85 × 10^–12^ C^2^ J^–1^ m^–1^, *N*_A_ is the Avogadro number, ρ is the density, and μ is the molecular dipole moment. ε_s_ and ε_∞_ denote the static and high-frequency dielectric permittivity, respectively.^17^ Herein, ε_∞_(*T*) was taken from the refractive index measurements ε_∞_ = 1.05*n* ^2^. The obtained temperature dependence of *g*_K_ is presented in the inset to Figure 2a. As can be seen, generally, the *g*_K_ value is much lower than unity over the entire examined temperature range. This indicates antiparallel orientations of permanent dipoles in the examined system, as visualized in Figure 2b. These data indicate that the postulated correlation between the high-frequency power law exponent of the dielectric loss peak and the Kirkwood correlation factor, *g*_K_, i.e, large values of β are associated with large values of *g*_K_,^18^ also does not work for the examined FOS.

**Figure 2:**
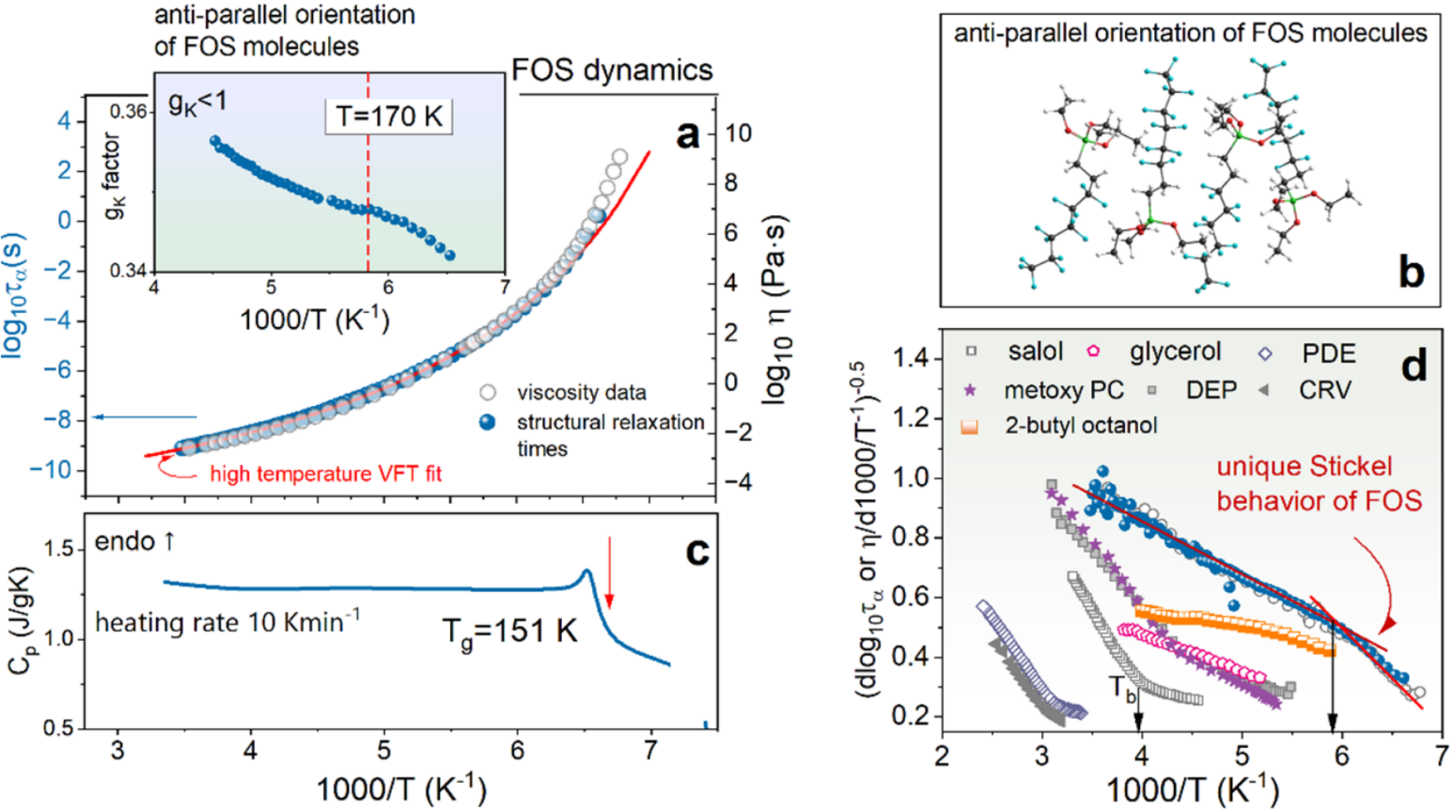
(a) Temperature dependence of structural relaxation times (left axis) and viscosity (right axis). The solid line presents the VFT fit of the high-temperature data. The VFT parameters obtained from parametrization of viscosity data are as follows: (high-temperature regime) log η_0_ = – 4.96 Pas, *D* = 7.616, *T*_0_ = 117.4 K; (low-viscosity range) log η_0_ = −2.06 Pas, *D* = 2.99, *T*_0_ = 132.2 K. Fragility parameter determined from equation *m* = 76 (high-temperature regime), *m* = 106 (low-temperature regime). **Inset:** temperature dependence of Kirkwood correlation factor. (b) Visualization of the antiparallel ordering of FOS molecules determined from the DFT method. (c) DSC thermogram of the examined system. (d) Temperature dependence of the Stickel operator for FOS and different glass-formers and associating liquids,^19^ i.e., conventional van der Waals glass-formers (DEP, diethyl phthalate; CRV, carvedilol; PDE, phenolphthalein dimethyl ether), and H-bonded associated liquids (2-butyl octanol).

Under these circumstances, the temperature evolution of the structural relaxation times of FOS is examined. The τ_α_ was determined directly from ε″(*f*) maxima and plotted vs 1000 *T* ^–1^ in Figure 2a. At first sight, log_10_ τ_α_ of FOS exhibits the behavior typical for glass-forming systems, i.e., it takes the non-Arrhenius temperature dependence that reaches 100s in the vicinity of calorimetric *T*_g_ (see Figure 2c). Furthermore, a single VFT function is not enough to describe the viscosity evolution over the entire temperature range. Therefore, according to standard practice, two VFT functions have been used to parametrize 12 decades of structural dynamics (see figure caption for VFT parameters). Upon careful examination of Figure 2c, it was discovered that the experimental data of FOS are more sensitive to temperature changes than predicted by the high-temperature VFT fit, which is in contrast to other glass-formers studied so far. The substantial temperature sensitivity of dynamic data close to *T*_g_ is reflected in a substantial increase of the fragility index *m* (calculated from the equation $m=\frac{DT_{0}T_{g}}{2.303{\left(T_{g}-T_{0}\right)}^{2}}$ ) when compared to a high-temperature regime (see Figure 2). Thus, a strong to fragile transition is observed in supercooled FOS. With the goal of determining precisely the temperature at which the FOS dynamics are significantly slow, the Stickel approach Φ = (*d* log_10_η/d 1000/*T*)^−0.5^ has been adopted. As presented in Figure 2d, the Stickel plot of FOS reveals two linear regions that intersect at around *T*_cross_ = 170 K. Moreover, in contrast to typical glass-forming liquids (e.g., salol, PDE, methoxy PC, CRV, or DEP), the slope of the Φ­(1000/*T*) dependence increases when we move from a high-temperature region to a low-temperature one. Notably, the characteristic crossover in the Stickel operator is observed not only in structural relaxation time behavior but also in the temperature evolution of shear viscosity (see Figure 2d). So far, such a unique behavior of the Stickel operator has been reported for strongly H-bonding systems (octanol, ethanol, note that the observed effect was much weaker than that determined for FOS; compare data for 2-butyl octanol and FOS in Figure 2d) and self-assembled ionic liquids. (see Figure 2d). The latter case was identified with dynamic evidence of the first-order liquid–liquid phase transition.^20–25^

However, for the examined glass form, there are no thermodynamic transformations above *T*_g_. As presented in Figure 2c, differential scanning calorimetry (DSC) thermogram obtained on reheating with a standard rate of 10 K min^–1^ over a wide *T* range revealed only a step-like change of heat capacity, indicating the liquid-glass transition (*T*_g_ = 150.9 K).

In the next step, temperature-dependent X-ray scattering and Raman experiments were performed to reveal the molecular origin of the described peculiarities in molecular dynamics. In the FOS diffractograms collected at room-temperature conditions (Figure 3), one can notice four characteristic maxima: one in the low scattering vector range (Q_1_ ∼ 0.4 Å^–1^), two subpeaks (Q_21_ ∼ 1 Å^–1^, Q_22_ ∼ 1.2 Å^–1^), and the last one, Q_3_, at ∼2.7 Å^–1^. The three peaks of the highest intensity are characteristic features of an amorphous halo. These “liquid-like” maxima are usually assigned to the nearest-neighbor intermolecular correlations. On the other hand, the prepeak observed at the low-q range (Q_1_) is a fingerprint of the nanoscale range ordering typical for associated liquids.^26^ As the temperature decreases, the amplitude of the prepeak rises while the intensities of Q_21_ and Q_22_ maxima decrease. The evolution of the peak position is also clearly detectable. At the SAXS scale, the decrease in the scattering vector upon cooling indicates an expansion in volume, akin to many metallic glasses. The fitting of WAXS data shows, in turn, the right shift of Q_2_ and Q_3_ peaks and their substantial separation, which reaches a maximum at *T* around 170 K (see the inset of Figure 3b). This is precisely in line with the peculiarities in structural dynamics described above. These results indicate the temperature-induced transition between the two preferred molecular alignments in the examined system. Since the subsequent heating brings opposite changes in the XRD pattern, long-range periodic order accompanying crystallization is excluded for FOS.

**Figure 3:**
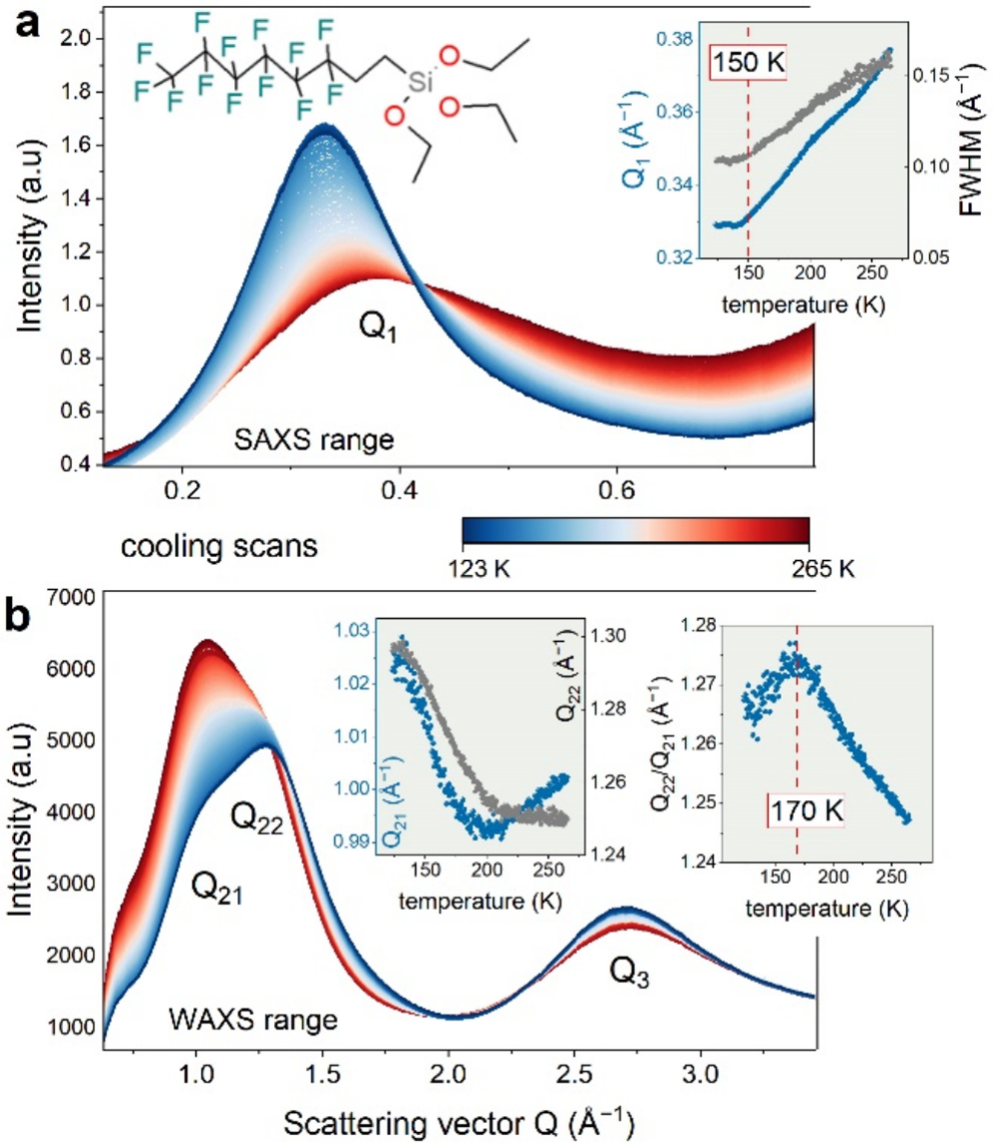
XRD pattern of supercooled and glassy FOS in SAXS (a) and WAXS (b) ranges. The insets show the evolution of peak position and the half-width at half-maximum (HWHM) of the peak during the cooling process.

The results of Raman experiments performed on cooling from 273 to 133 K are presented in Figure 4. There are numerous bands in the Raman spectra of FOS including asymmetric and symmetric vibrations of ethyl groups, stretching CC (1050 cm^–1^), and various deformation modes such as wagging (w), twisting (t), and rocking (r) of CH_3_ (1460, 1375, and 950^r^ cm^–1^), CH_2_ (1345^w^, 1215^t^, 1125^r^, and 830 cm^–1^), and stretching and deformation of the silyl fragment with (OSi)­CCH and (SiOC)C modes (1030, 960, and 640 cm^–1^). Additionally, due to the head-chain structure of the FOS molecule, the stretching and deformation modes of CF_3_–CF_2_– (1329, 745, and 715 cm^–1^), CF_2_ (1220 cm^–1^), and CF_3_ (660 cm^–1^) bands are activated in the lower-frequency range (Figure 4a,b). To explain the conformational order of FOS molecules, temperature-dependent alterations in the spectral pattern, such as integrated intensity or band position, were analyzed. As displayed in the insets to Figure 4, the integrated intensity of bands related to the triethoxysilane moiety and fluorocarbon chain exhibits a nonmonotonic behavior on cooling, with an apparent kink around 170 K. This behavior suggests the reorganization of FOS molecules similar to that originating from the liquid–liquid transition in ionic liquids with long alkyl chains. However, unlike the cationic structure, sheet-like molecular alignments in antiparallel configurations are observed.^27–29^ This hypothesis is supported by the shift of head-related individual bands toward higher frequencies (see insets of Figure 4), indicating the formation of a 3D network configuration with molecular ordering and nanosegregation due to the weak H-bond between fluorine and proton of the CH_2_ group. On the other hand, despite the internal reorganization of the ethyl group CH_3_, OSiOC–, C_2_H_4_–, and CCF_2_-related bands show negligible (within the statistical error) conformational sensitivity of band shifts without a crossover in the temperature-dependent profile. As a result, it is reasonable to believe that variations in the ratio between nonordered and antiparallel molecular configurations within sheet-like structures are the cause of the nanosegregation seen within the temperature range of 190–150 K. Below the glass transition temperature, an opposite trend in integrated intensities was observed for all FOS bands, indicating the freezing of local motions of the entire molecules in the newly formed conformations.

**Figure 4:**
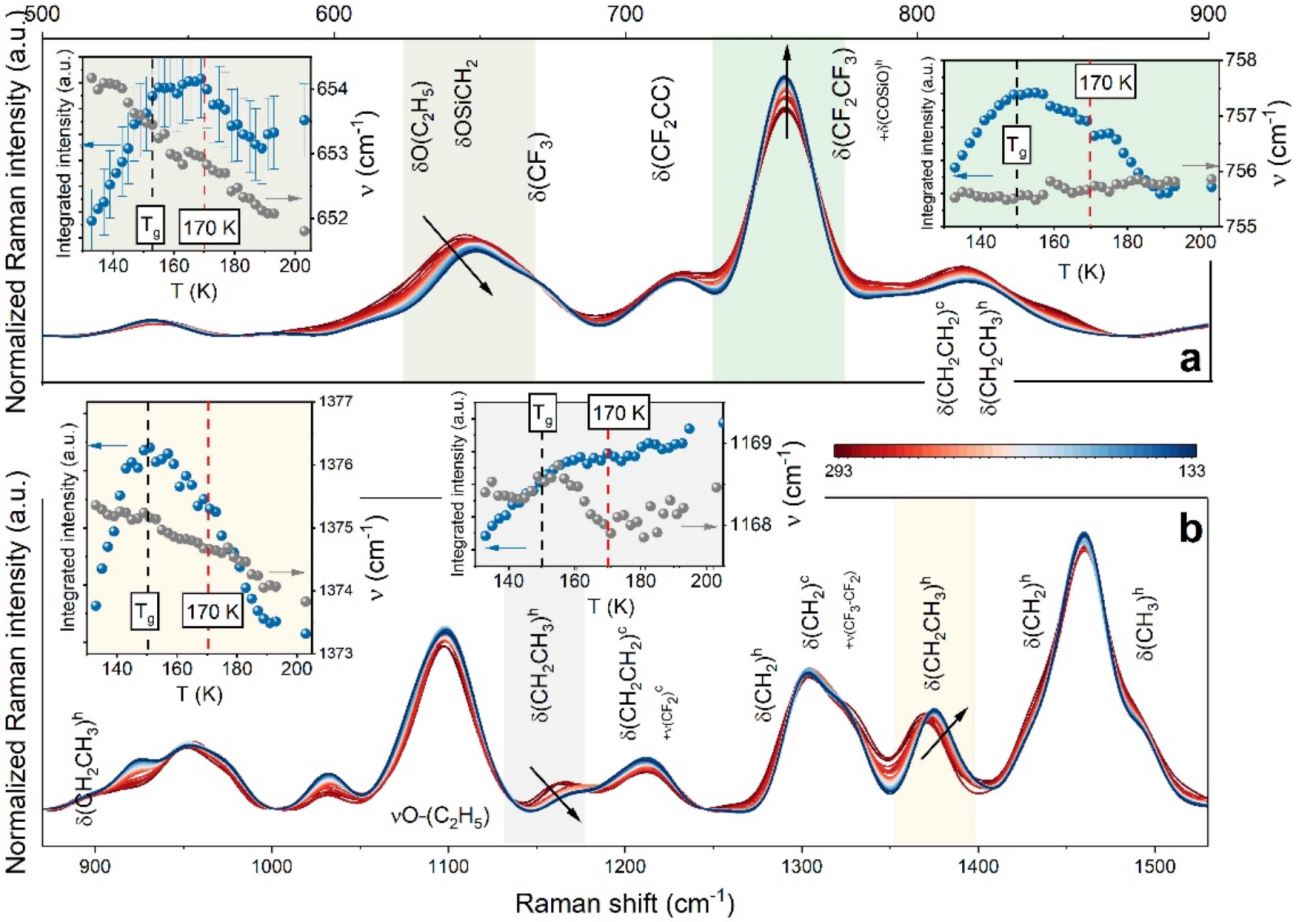
Temperature-dependent Raman spectra of FOS within the ranges of 500–900 cm^–1^ (a) and 850–1550 cm^–1^ (b) summarized with the integrated intensity and band shift of the δ­(CH_2_CH_3_)-related band and δ­(C_2_H_5_)-related band, within the polyfluorinated alkane straight chain, respectively, and the δO­(C_2_H_5_)-related band and the δ­(COSiO)-related band within the triethoxysilane fragment, respectively.

## Conclusions

In summary, we employed four experimental techniques to investigate the relation between the structure and dynamics in the deeply supercooled state of the polar glass-former, revealing molecular self-assembly. The examined system transforms into the amorphous form at 150 K. However, 20 K above the liquid-glass transition, it undergoes substantial structural and dynamic changes. Specifically, at 170 K, the molecular dynamics markedly slow down, i.e., logτ_α_ and logη vs 1000/T changes the behavior from strong (at *T* > 170 K) to fragile (at *T* < 170 K), which is accompanied by a well-pronounced Stickel crossover and a decrease in the Kirkwood correlation factor. The latter suggests antiparallel alignment of mobile dipoles that explain the low value of a static dielectric constant. At the same temperature, noticeable changes in the XRD diffraction pattern and Raman spectra occur, thereby confirming the existence of some long-lived local favorite structures due to the molecular self-assembly of fluorocarbon chains. Since the observed structural and dynamical changes are fully reversible and do not bring a calorimetric fingerprint (DSC thermogram shows only *T*_g_ on cooling and subsequent reheating), a negligible difference in entropy between LFS and normal liquid structures is expected.
